# Editorial: New lights Through Old Windows: Metformin and Derivatives as Anti-Cancer Treatments

**DOI:** 10.3389/fphar.2022.889642

**Published:** 2022-04-26

**Authors:** Aiman Moldasheva, Vladimir Surov, Mohamad Aljofan

**Affiliations:** Department of Biomedical Sciences, School of Medicine, Nazarbayev University, Nur-Sultan, Kazakhstan

**Keywords:** metformin, anticancer, drug repurposing, repositioning, rescue, drug screening, autophagy

The current theme issue is intended to focus on the advances and the development of drug repurposing and strategies for cancer treatment. The issue contains the contributions from researchers studying various aspects of drug repurposing and screening for anticancer management with a particular interest in metformin (dimethylbiguanide) as well as other small marketed drugs.

Metformin is the recommended first line of treatment of type 2 Diabetes Mellitus. Recently, metformin is gaining international interest for its potential use to treat/prevent different types of cancer, cardiovascular disease, ageing, and neurological disorders. Several studies reported the potential anticancer activity of metformin that promoted the initiation of tens of clinical trials that aimed to investigate its potential against endometrial, prostate, pancreas, lung and breast cancer. Nonetheless, uncertainties still exist with respect to understanding the cellular mode of action of metformin, its antiproliferative ability as well as the effective antiproliferative concentration.

In their article “Metformin Adjunct With Antineoplastic Agents for the Treatment of Lung Cancer: A Meta-Analysis of Randomized Controlled Trials and Observational Cohort Studies” (Luo et al.) that evaluated the scientific literature on the efficacy of the combinational use of metformin and anticancer agents on lung cancer survival in both diabetic and non-diabetic patients. Briefly, the study is a comprehensive meta-analyses of randomized controlled trials (RCTs) and observational cohort studies with two outcome indicators, namely overall survival (OS) and progression-free survival (PFS). The clinical evidence obtained from the current analysis suggested that metformin, in combination with antineoplastic agents, may improve the PFS and OS compared to standard antineoplastic agents alone. While this finding is in line with our previously published work that metformin may provide synergistic effect to chemotherapy (Aljofan and Riethmacher, 2019), a number of studies suggested that metformin could be used, on its own, as a monotherapy for the management of cancer (Amaral et al., 2018; He et al., 2018; Mallik and Chowdhury, 2018; Tan et al., 2018). Though, the precise anticancer mechanism of metformin remains undetermined, a number of potential mechanisms were reported with the majority revolving around the activation of AMP-activated protein kinase (AMPK) pathway (Aljofan and Riethmacher, 2019), such as the induction of autophagy as a result of inhibition of mammalian target of rapamycin as a result of AMPKactivation (Luo et al.).

Interestingly, another study in this theme titled “The Disulfiram/Copper Complex Induces Autophagic Cell Death in Colorectal Cancer by Targeting ULK1” (Hu et al.) claimed that disulfiram complexed with Copper (DSF/Cu) has a potential in the treatment of colorectal cancer. The study claimed that DSF/Cu inhibits cell viability of colorectal cancer cells in a dose dependent manner both *in vitro* and *in vivo* (subcutaneous xenograft mice model). The authors suggested that the DSF/Cu complex induced inhibition of cell viability was due to cellular autophagy rather than apoptosis. To confirm the potential mechanism, the authors employed a (CRISP-Cas9)library screening to identify genes, expression of which was either up or down regulated by DSF/Cu complex exposure. Screening resulted in identification of five –autophagy related genes (ULK1, ATG16L2, ATG12, LAMP3, and PIK3C3), out of which expression of ULK1 was significantly upregulated. ULK1 is a kinase that participates in autophagosome assembly. Effect of DSF/Cu treatment on induction of autophagy was abolished in ULK1 knockdown cells, which was showed by suppressed expression of LC3 (marker of autophagosomes) after DSF/Cu treatment of ULK1 knockdown cells. This suggests that autophagy can at least partially play role in DSF/Cu induced cytotoxicity. Moreover, DSF/Cu induced cytotoxicity could be partially overcome by pretreatment of cells with autophagy inhibitor—choloroquine (CQ).

Nevertheless, activation of apoptosis or programmed cell death is considered as a major drug target for most anti-cancer therapies, particularly breast cancer. While chemotherapy is considered as the main treatment for breast cancer, there is a poor overall response in breast cancer patients due to the development and activation of anti-apoptotic systems that facilitate the escape of drug-induced apoptosis (Takagi et al., 2015). In their contribution to this issue titled “Triclabendazole Induces Pyroptosis by Activating Caspase-3 to Cleave GSDME in Breast Cancer Cells” Yan et al., described the potential anticancer effect of triclabendazole, a benzimidazole compound, indicated for the treatment of fascioliasis. Interestingly, the death of breast cancer cells (MCF-7) upon exposure to triclabendazole displayed typical signs of pyroptosis including annexin V staining, regulating the apoptoic protein levels of Bax, Bcl-2, and enhanced cleavage of caspase-8/9/3/7 and PARP. Furthermore, cellular treatment with triclabendazole resulted in an enhanced cleavage of gasdermin E (GSDME), which is a key molecule in the pyroptosis process, indicating that triclabendazole is a potent inducer of pyroptosis.

Drug repurposing involves finding new therapeutic benefits of marketed drugs rather than searching for entirely new therapeutic agents. The process has become especially important for developing anticancer therapies, as the majority of currently available oncology drugs have numerous side effects, low efficacy and high cost. In this themed issue, we assembled a collection of various studies that investigated drugs with potential anticancer activity including metformin, ivermectin, triclabendazole, and disulfiram copper complex. While these drugs appear to have different potency and mechanism of actions, they all showed positive anticancer activity warranting further research to evaluate their potential repurposing for anticancer uses.
